# Multiple Classification of Brain MRI Autism Spectrum Disorder by Age and Gender Using Deep Learning

**DOI:** 10.1007/s10916-023-02032-0

**Published:** 2024-01-22

**Authors:** Hidir Selcuk Nogay, Hojjat Adeli

**Affiliations:** 1 Electrical and Energy Department, Bursa Uludag University, Bursa, Turkey; 2 Departments of Biomedical Informatics and Neuroscience, College of Medicine, The Ohio State University Neurology, 370 W. 9th Avenue, Columbus, OH 43210 USA

**Keywords:** ASD, Multiple classification, CNN, CED, Data augmentation, GSO, sMRI

## Abstract

The fact that the rapid and definitive diagnosis of autism cannot be made today and that autism cannot be treated provides an impetus to look into novel technological solutions. To contribute to the resolution of this problem through multiple classifications by considering age and gender factors, in this study, two quadruple and one octal classifications were performed using a deep learning (DL) approach. Gender in one of the four classifications and age groups in the other were considered. In the octal classification, classes were created considering gender and age groups. In addition to the diagnosis of ASD (Autism Spectrum Disorders), another goal of this study is to find out the contribution of gender and age factors to the diagnosis of ASD by making multiple classifications based on age and gender for the first time. Brain structural MRI (sMRI) scans of participators with ASD and TD (Typical Development) were pre-processed in the system originally designed for this purpose. Using the Canny Edge Detection (CED) algorithm, the sMRI image data was cropped in the data pre-processing stage, and the data set was enlarged five times with the data augmentation (DA) techniques. The most optimal convolutional neural network (CNN) models were developed using the grid search optimization (GSO) algorism. The proposed DL prediction system was tested with the five-fold cross-validation technique. Three CNN models were designed to be used in the system. The first of these models is the quadruple classification model created by taking gender into account (model 1), the second is the quadruple classification model created by taking into account age (model 2), and the third is the eightfold classification model created by taking into account both gender and age (model 3). ). The accuracy rates obtained for all three designed models are 80.94, 85.42 and 67.94, respectively. These obtained accuracy rates were compared with pre-trained models by using the transfer learning approach. As a result, it was revealed that age and gender factors were effective in the diagnosis of ASD with the system developed for ASD multiple classifications, and higher accuracy rates were achieved compared to pre-trained models.

## Introduction

ASD is a neurodevelopmental disease that occurs in early childhood and is characterized by communication disorders and difficulties in socialization in children [1, 2]. There has been an increase in the incidence of Autism Spectrum Disorder over the years, and while one in every 150 children in America was autistic in 2000, it is reported that one in every 54 children has autism in 2020 [3, 4].

Despite an extensive range of signs of ASD [5], a complication that prolongs the diagnosis process is the high rate of comorbidity. The comorbidity problem in children with ASD means an extra disability like a vision problem or another health problem [6]. A study revealed that 88.5% of children diagnosed with autism had at least one of the neurodevelopmental disorders such as attention deficit hyperactivity disorder (ADHD), intellectual disability and developmental coordination disorder [7]. The incidence of autism is higher in boys than in girls [8]. Although the reason for this is not clear, hypotheses such as Extreme Male Brain, Female Protective Effect, and Female Autism Phenotype are being studied [9]. The lack of a known cure for autism, the long diagnosis and treatment process [10], and the high degree of comorbidity all indicate that more scientific work is needed on autism [11]. There is an important need to study the influence of age and gender factors on ASD diagnosis and to evaluate the possibility that multiple classifications, including age and gender, may contribute to the rapid early diagnosis of ASD. Recent genetic works show that ASD occurs differently between males and females and between youths and adults [12]. Artificial intelligence and machine learning (ML) techniques [13, 14] such as DL provide fresh opportunities to discover biomarkers for diagnosis of ASD taking into account factors like age and gender that affect ASD, to shorten the diagnostic process of ASD, to avoid subjective opinions of different doctors and possibly reach a definitive diagnosis [15–17].

DL techniques have found extensive application in medical and neurological fields such as seizure detection [18], seizure prediction [19–21], epilepsy diagnosis and classification [22, 23], autism [24–27], optimization of neuroprosthetic vision [28], post-stroke rehabilitation with motor imagery [29], sentiment analysis [30], emotion recognition [31, 32], patient-specific quality assurance [33], classification of the intracranial electrocorticogram [34], brain-computer interface (BCI) for discriminating hand motion planning [35], dyslexia biomarker detection [36–38], and many other fields such as mobile robots [39], drone-based water rescue and surveillance [40], and structural health monitoring in recent years [41–43].

The design and effectiveness of a DL method for diagnosing ASD varies according to the data set. The data set can be numeric or two-dimensional graphical, or visual data. Numerical data can be behavioral [44, 45], eye-following [46], or fingerprint data [47–49], converted into numerical data by pre-processing. Optical data are brain structural magnetic resonance scanning images (sMRI) or brain functional magnetic resonance scanning images (fMRI). Using numerical or visual data to train an ML algorithm for ASD diagnosis is ordinarily possible by determining the distinguishing features or using an automated feature extraction technique [50–52]. These features may be structural gray matter (GM) values acquired from cortical thickness (CT) [53–55], GM density (GMd) values from voxel-based morphometry (VBM) [56], diffusion-weighted imaging (DWI) [fractional anisotropy (FA)] in white matter (WM)) microorganism changes [57], connectivity matrices [58], parameters from network analysis [59–61], and resting/duty state fMRI information [62, 63]. However, if a type of DL known as convolutional neural network (CNN) is utilized, direct classification is performed because feature extraction is done automatically. This is known as end-to-end deep learning [64]. For this reason, the CNN method is employed in this research as the most suitable method for rapid diagnosis of ASD.

In the study, the influence of a certain age range and gender on the diagnosis of ASD is examined by performing multiple classifications of ASD based on age and gender. A DL system has been introduced that can diagnose ASD for certain age ranges and gender. The advantages and differences of the current research compared to previously-reported research on ASD diagnosis, binary classification, and/or multiple classification works can be listed as follows. First, multiple classifications, including age and gender, were performed in this study, and to the best of the authors’ knowledge, this has never been done before. Second, compared to other works that employ a DA method, the number of image data in this study is huge and acquired from different brain regions. This is advantageous in terms of the generalizability of the models. Third, CNN was designed from scratch and utilized as a system element in this study. Thus, feature extraction is done automatically. Fourth, using a transfer learning (TL) method, today’s popular pre-trained models were trained and tested with the same data set.

The following sections are organized as follows. In the next section, works on ASD classification using brain MRI images, which also considered other factors like age and gender, are discussed. The third section explains the techniques and materials utilized in the study. In the fourth section, metrics used to evaluate the performance of the study are presented. The fifth section reports the numerical experimental results acquired from the study. The paper ends with discussions and a conclusion.

## Related works

Although multiple classifications are more informative for ASD diagnosis works using brain sMRI scans, researchers have not studied them due to their complexity and difficulty in achieving high accuracy rates. As a result, the authors could not find a CNN model trained with brain sMRI images that could perform quadruple and octal classification, including gender and age factors, for ASD diagnosis. Therefore, in this study, multiple classifications were made through binary pairings such as F-ASD and F-TD, M-ASD and M-TD (F represents female, M represents male, and TD represents typical development) using brain MRI images. The quadruple classification was made using only gender, another quadruple classification using only age range, and the octal classification using both gender and age factors.

In a multiple binary classification study of ASD conducted by [12], they created separate groups like ‘adolescents-F (< 18years)’ - ‘adolescents-M(< 18years)’ and ‘adults-F(> 18years)’ - ‘adult-M(> 18years)’ [12]. They investigated which group could be diagnosed with ASD with the highest accuracy rate by making separate binary classifications using by Extended Metacognitive Radial Basis Function Neural Classifier (EMcRBFN) method, which is trained and tested by sMRI data. They found that ASD can be detected more accurately in women (81%) than in men (60%). In [65], it investigated the impact of gender factors on the diagnosis of ASD in multiple binary classifications. In their study with the Support Vector Machine (SVM) method, they obtained an accurate prediction rate of 69% for the ASD-F (female) group and 66% for the ASD-M (male) group. However, the data set was limited to the 18–49 age group [65]. In [66], it employed DL trained with brain fMRI scans and performed binary ASD classification reporting a 70% accuracy [66]. In another DL study dealing with the age factor, they were able to diagnose ASD in the 2-year-old group with an accuracy rate of 76.24% using the “Multi-Channel Convolutional Neural Network” (MC-CNN) [67]. In [68], they performed a binary classification of ASD and reported an accuracy rate of 65.69% deep belief network (DBN) model [68]. In [69], they diagnosed ASD with 90.39% accuracy in binary classification using a DL algorithm trained with brain sMRI scans of participators whose mean age was 15 [69].

## Materials and methodology

### Dataset

The ABIDE database, an international professional database made available on March 27, 2017, was used to train and test the models in the study [70]. Detailed information about ABIDE can be obtained from http://fcon_1000.projects.nitrc.org/indi/abide/. The data in the ABIDE database consist of data collected from 29 different sites shown in Table 1. T1 weighted sequence and sMRI of 2248 participators, 1072 ASD and 1176 TD gathered from 29 locations from ABIDE, constituted first repository of the study called Data1. All images in the repository were scanned for clarity one by one. After the sharpness scan, a sum of 1831 image data, 938 ASD and 893 TD, were used as Data1 in this study. No coloration or any application affecting discrimination was made on any image data. For the three multi-classification CNN models utilized in the study, the data were distributed for all three models, as shown in Table 2, before pre-processing.

**Table 1:** Distribution of the data utilized in this research

**SITE**	**ASD**		**TD**		**ALL**
	**Ages**				
	**5–17**	**18–65**	**5–17**	**18–65**	
STANDFORD	20	0	20	0	40
KKI	77	0	188	0	265
KUL	5	23	0	0	28
LEUVEN	16	13	21	14	64
UCD	19	0	14	0	33
OHSU	51	0	70	0	121
MAXMUN	9	15	6	26	56
UCLA	81	0	68	0	149
BNI	2	25	2	27	56
CALTECH	1	18	2	17	38
EMC	27	0	27	0	54
GU	51	0	54	0	105
IP	17	4	10	23	54
NYU	134	20	104	31	289
PITT	18	12	15	12	57
SDSU	46	0	47	0	93
TRINITY	16	8	15	10	49
UM	80	0	89	3	172
UPSM	17	1	15	2	35
YALE	28	0	28	0	56
SU	21	0	21	0	42
OLIN	14	6	10	6	36
ETH	4	7	3	21	35
TCD	18	3	16	5	42
IU	2	17	0	19	38
ONRC	5	18	1	28	52
USM	36	38	26	32	132
CMU	0	14	0	13	27
SBL	0	15	0	15	30
ALL Sites	815	257	872	304	2248

**Table 2:** Summary of the data sets

**Datasets**	**Class** **Number**	**Groups**	**Size**	**Total** **Size**	**Gender**	**Age range**
Data1	1	ASD + f	127	1831	Female	-
	2	ASD + m	811		Male	-
	3	TD + f	209		Female	
	4	TD + m	893		Male	
Data2	1	ASD 5–17	648	1831	-	5–17
	2	ASD 18–65	290		-	18–65
	3	TD 5–17	594		-	5–17
	4	TD 18–65	299		-	18–65
Data3	1	ASD 5–17 f	96	1831	Female	5–17
	2	ASD 5–17 m	552		Male	5–17
	3	ASD 18–65 f	31		Female	18–65
	4	ASD 18–65 m	259		Male	18–65
	5	TD 5–17 f	157		Female	5–17
	6	TD 5–17 m	437		Male	5–17
	7	TD 18–65 f	52		Female	18–65
	8	TD 18–65 m	247		Male	18–65

### Data pre-processing

**Fig. 1:**
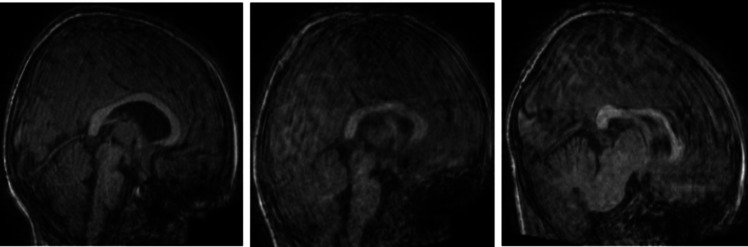
Examples of eliminated sample images

The raw data were subjected to a three-step pre-processing. In the first pre-processing step, the unclear images were eliminated. Figure 1 shows examples of vague images.

**Fig. 2:**
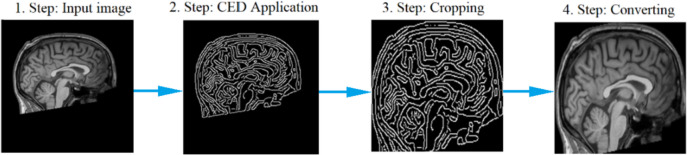
The second step of the data pre-processing

In the second pre-processing step, the Canny Edge Detection (CED) algorithm subjected each image data to edge detection. The new image acquired after CED processing is cropped from the determined edges, and the lost area is minimized. Figure 2 describes the second step of data pre-processing, including the CED processing.

**Fig. 3:**
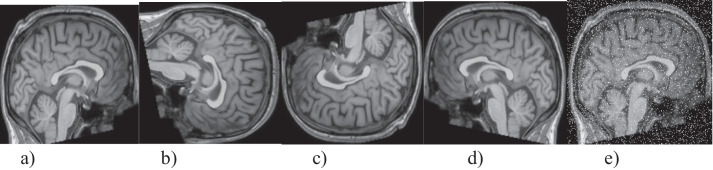
**a** The raw image, **b** rotating by 90 degrees, **c** rotating by 180 degrees. **d** right/left flip, **e** 5% salting to the image

In the third step of the pre-processing, each image was subjected to DA by right-left flip, 90^o^ right rotation, 180^o^ right rotation, and 5% salting, magnifying the dataset fivefold. Figure 3 shows the application of the planned DA technique for only one sample image.

### Proposed CNN models

#### Optimal hyper-parameter selection

**Fig. 4:**
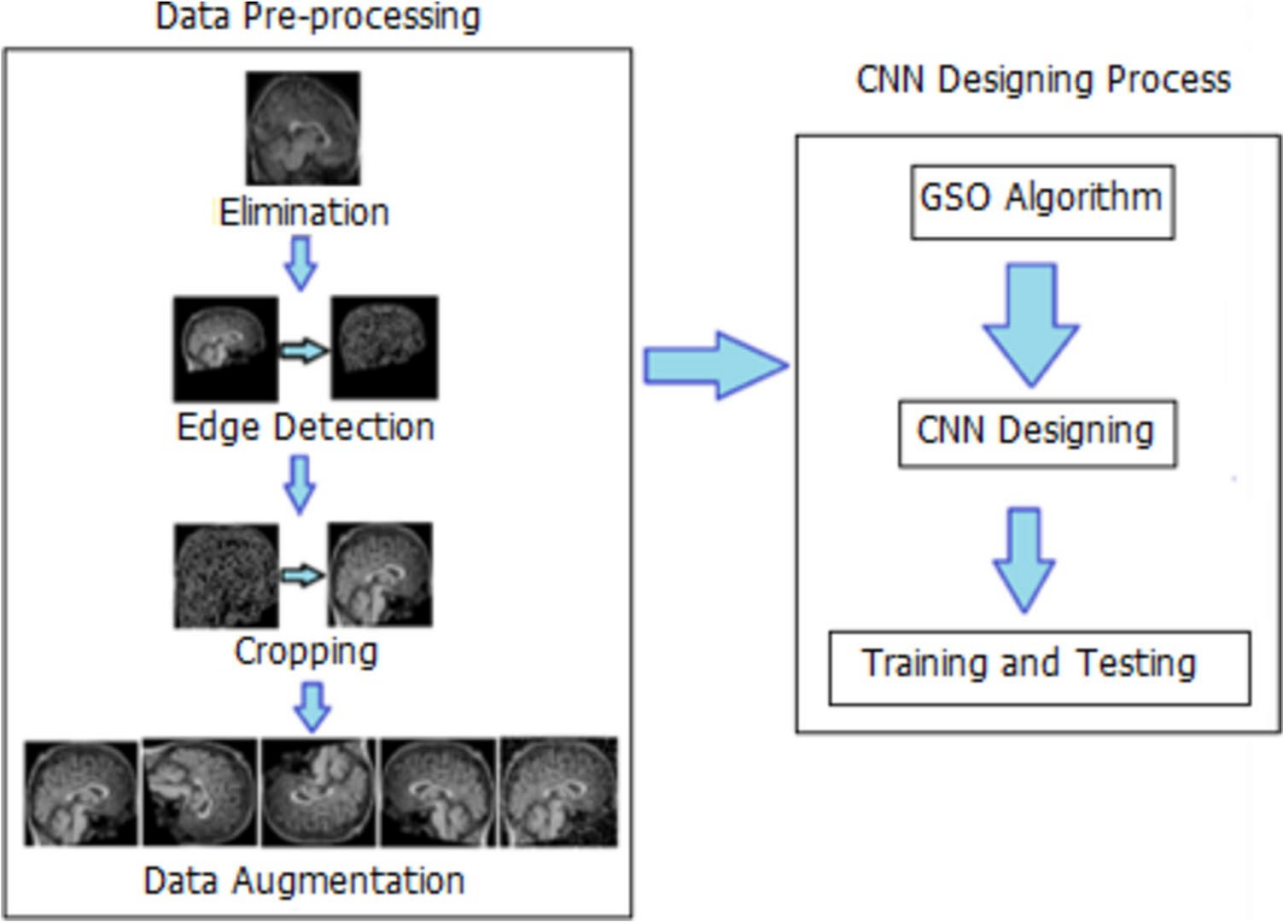
Schematic representation of the proposed system

Three DL models were designed as part of the system developed in this study. For each model, the GSO algorithm was utilized to decide the most optimal hyperparameters among the limit values determined in Table 3 [71]. After pre-processing the data set, each model was tested with a randomly selected 20% of the ready-to-utilize data. From top to bottom, the first five rows of hyperparameters in Table 3 are about the architecture of the CNN models, and the next five are about fine-tuning each architecture. In Fig. 4, the system designed in the study is described schematically.

**Table 3:** Hyper-parameters and value ranges

	**Hyper-parameters to optimize**	**Value ranges**
1	Number of Convoluiton layer	[1, 2, 3, 4, 5, 6, 7, 8]
2	Number of Maxpooling layer	[1, 2, 3, 4, 5, 6, 7, 8]
3	Number of FC layers	[1, 2, 3, 4]
4	Number of filters	[16, 24, 32, 48, 64, 96]
5	Filter sizes for conv and pooling	[2, 3, 4, 5, 6, 7]
6	Padding	[0, 1, Same]
7	Stride	[1, 2, 3]
8	L2 regularization	[0.0001, 0.0005, 0.001, 0.005]
9	Momentum	[0.70, 0.75, 0.80, 0.85, 0.9, 0.95]
10	Mini-batch size	[8, 16, 32, 64, 128]
11	Learning rate	[0.0001, 0.0003, 0.0005, 0.001, 0.003, 0.005]
12	Activation function	ReLu, Leaky Relu, ELU, SELU

#### Convolution and pooling

The convolution operation is the processing of acquiring the **B** output matrix as a result of filtering the **A** image matrix entering a CNN model with the **K** filter matrix, as shown in Eq. (1). The resulting output matrix **B** is smaller than the input matrix **A**. During the filtering processing, the filter matrix **K** on matrix **A** can be shifted as much as the shift step (*stride*). In some strategies, resizing the output matrix to the same size as matrix **A** may be desirable. In this case, it can be brought to the same size as the **A** matrix by filling the blank parts of the **B** matrix with the number zero. This processing is called *padding*. In addition, before the convolution operation, pooling, which is a sub-operation of the convolution operation, is performed to reduce overfitting. In the pooling processing, the input matrix of the pooling layer is filtered by the selected filter matrix on the principle of mean or maximum values [72]. With Eq. 2, the size of the output matrix is obtained as a result of the filtering used in both pooling and convolution operations [72].

1 $$B_{ij}={\left(A\ast K\right)}_{ij}={\textstyle\sum_{f=0}^{n_k-1}}{\textstyle\sum_{h=0}^{n_k-1}}A_{i+f,j+h}K_{i+f,j+h}$$

2 $${n}_{B}= \lfloor\frac{{n}_{A}+2p-{n}_{K}}{s}+1\rfloor$$

#### Softmax and classification

The cross-entropy loss is calculated during the classification process. Softmax function is the layer before the classification layer. Multiclassification is performed as probabilistic in the Softmax layer. The softmax function for the multiple classifications is expressed as follows [73].

3 $${y}_{r}\left(x\right)=\frac{\text{e}\text{x}\text{p}\left[{a}_{r}\left(x\right)\right]}{{\sum }_{j=1}^{k}\text{e}\text{x}\text{p}\left[{a}_{j}\left(x\right)\right]}$$

In Eq. (3); $0\le {y}_{r}\le 1$, $\sum _{j=1}^{k} {y}_{j}=1$, and ${a}_{r}$ is the conditional probability of the given *r* class sample [73].

#### Designing processing of the proposed models

**Fig. 5:**
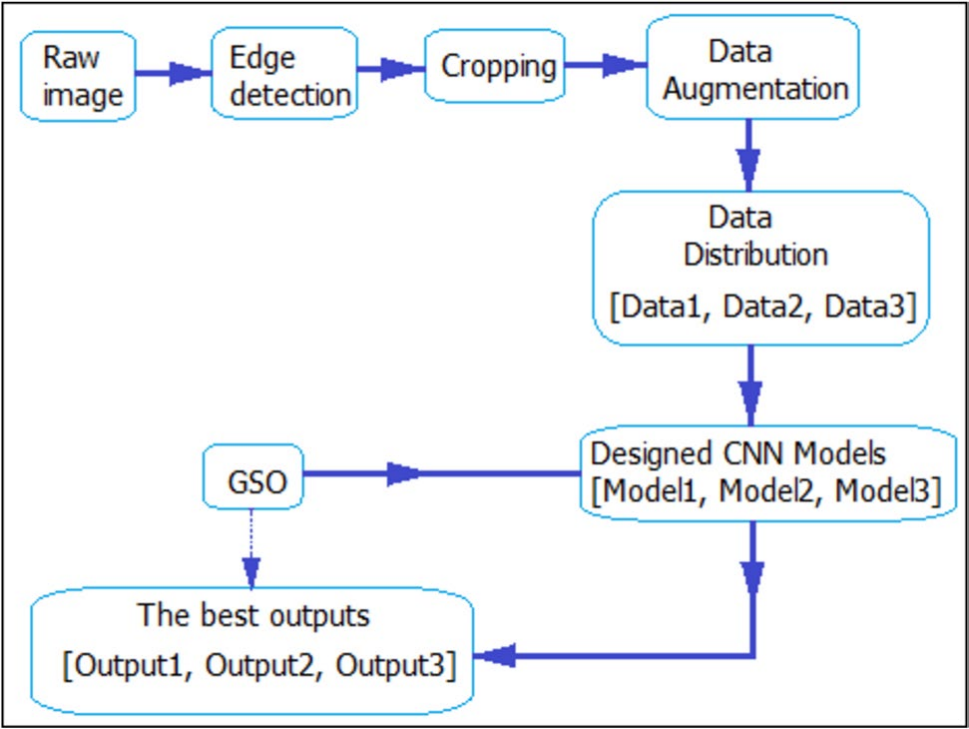
Flow chart of the proposed system

Three multiple classifications were performed using the system designed within the scope of this study. First, the acquired brain sMRI image data were pre-processed. After pre-processing, the data were divided into three separate data sets, taking into account age, gender, and both. Grid search optimization (GSO) algorithm was utilized to design the CNN models to be trained with these three separate data sets from scratch to achieve optimal hyperparameters and the highest accuracy rate. The flow diagram of the designed system is illustrated in Fig. 5.

**Fig. 6:**
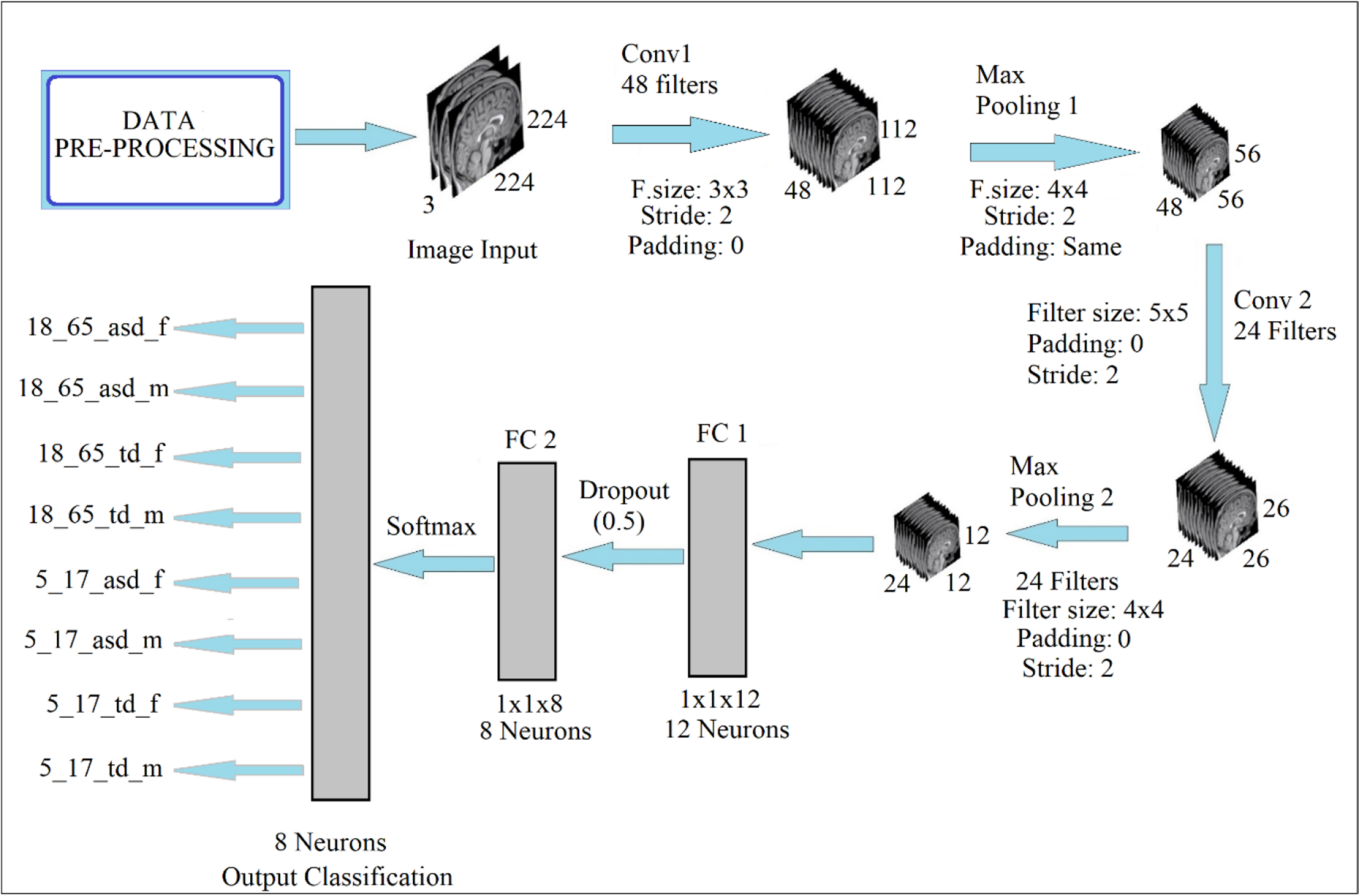
The architecture of the designed Model 3

In the study, the dimensions of the input data matrix is chosen as 224 × 224 regardless of any criterion. Input image sizes are not contained in the GSO algorithm. The study utilized Model 1, Model 2, and Model 3 CNN model names for Data 1, Data 2, and Data 3, respectively. Table 4 shows the hyperparameters decided due to the GSO for each model and the structures of the CNN models thus designed. Figure 6 shows the architectural scheme in which Model 3 is utilized in the developed system.

**Table 4:** Optimal hyper-parameters of the proposed CNN models

		**Value**		
	**Hyper-parameters**	**Model 1 (Gender)**	**Model 2 (Age)**	**Model 3 (Both)**
1	Number of Convoluiton layer	7	6	2
2	Number of Maxpooling layer	7	6	2
3	Number of FC layers	2	2	2
4	Number of filters [Conv1, Pool1, Conv2, Pool2, Conv3, Pool3, Conv4, Pool4, Conv 5, Pool 5, Conv 6, Pool6, Conv7, Pool7…]	[48, 48, 24, 24, 24, 24, 24, 24, 16 16,16, 16, 16, 16]	[48, 48, 32, 32, 24, 24, 24, 24, 16, 16, 16, 16]	[48, 48, 24, 24]
5	Filter sizes [Conv1, Pool1, Conv2, Pool2, Conv3, Pool3, Conv4, Pool4, Conv 5, Pool 5, Conv 6, Pool6, Conv7, Pool7 …]	[3, 4, 3, 3, 5, 4, 5, 5, 3, 3, 3, 3, 5, 4]	[4, 3, 4, 3, 5, 4, 3, 5, 4, 4, 4, 4]	[2, 4, 5, 4]
6	Padding [Conv1, Pool1, Conv2, Pool2, Conv3, Pool3, Conv4, Pool4, Conv 5, Pool 5, Conv 6, Pool6, Conv7, Pool7 …]	[0, 0, 1, 0, 0, 0, 0, 0, 1, 0, 0, 0, 0, 1]	[0, 0, 0, 0, 0, 1, 0, 0, 0, 0, 0, 0]	[0, same, 0, 0]
7	Stride [Conv1, Pool1, Conv2, Pool2, Conv3, Pool3, Conv4, Pool4, Conv 5, Pool 5, Conv 6, Pool6, Conv7, Pool7 …]	[1, 1, 2, 1, 1, 1, 1, 1, 2, 2, 2, 1, 2, 1]	[1, 1, 1, 1, 1, 1, 1, 1, 2, 2, 2, 2]	[2, 2, 2, 2]
8	L2 regularization	0.0001	0.0001	0.0001
9	Momentum	0.9000	0.9000	0.9000
10	Mini-batch size	32	32	32
11	Learning rate	0.0001	0.0001	0.0002
12	Activation function	ReLu	ReLu	ReLu

## Performance metrics

Utilizing the loss function shown in Eq. (4), the network continues to be trained throughout the training processing of the network until the loss values calculated for each iteration reach their minimum value.

4 $$Loss=-{\textstyle\sum_{i=1}^N}{\textstyle\sum_{j=1}^K}t_{ij}\text{l}\text{n}\;y_{ij}$$

In Eq. (4), we have N samples represented by *t*_*ij*_, where each sample I belongs to one of the K classes, and the corresponding output *y*_*ij*_ is assigned to sample *j* of class *I* [73]. Equation (5) shows the accuracy rate as another performance criterion [73].

5 $$Accuracy=\frac{Total\;correct\;prediction\;labels}{Total\;Equation\;Number\;of\;real\;labels}\times 100$$

## Experimental results

**Fig. 7:**
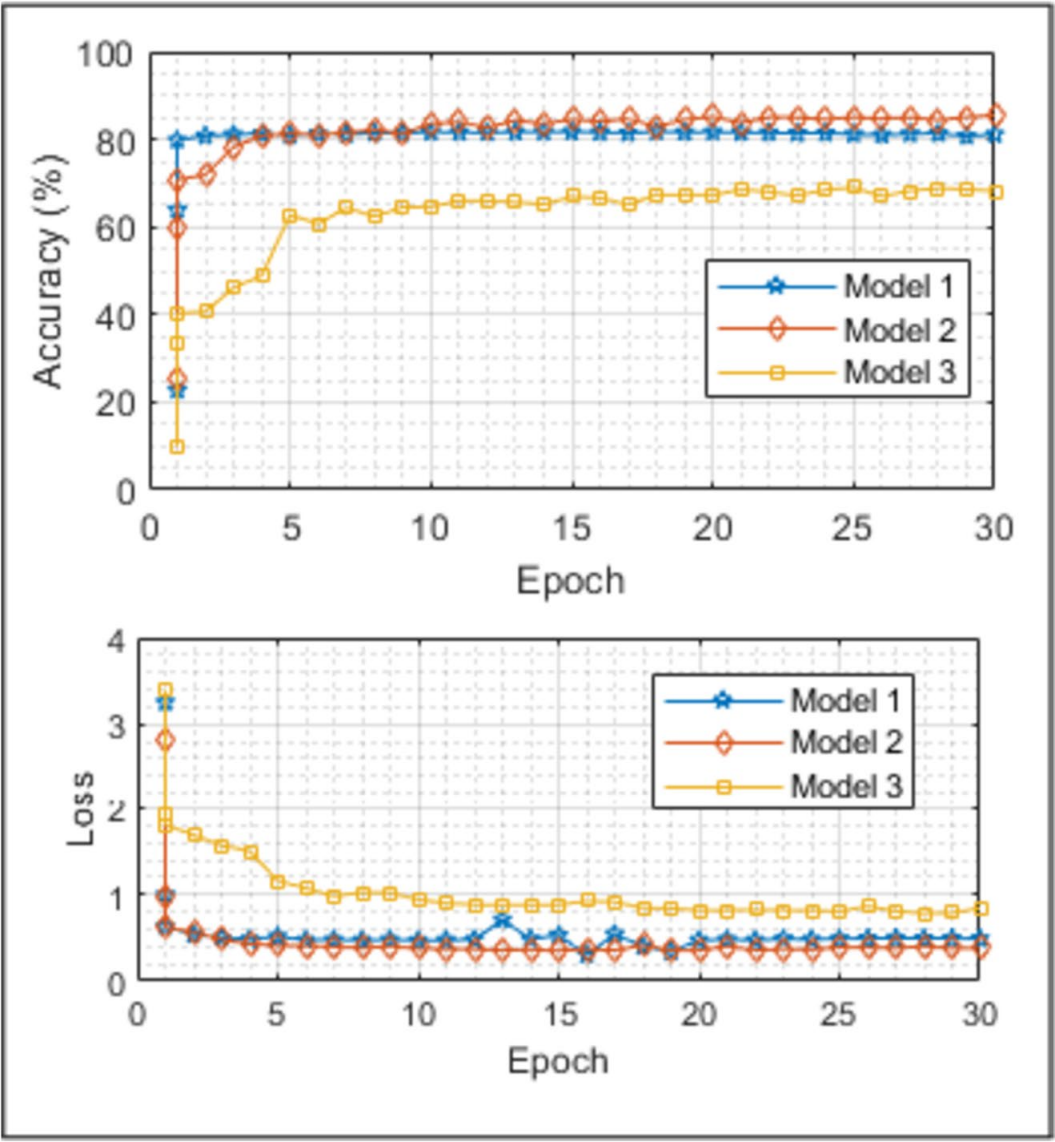
Accuracy and Loss curves for all three classifications

**Fig. 8:**
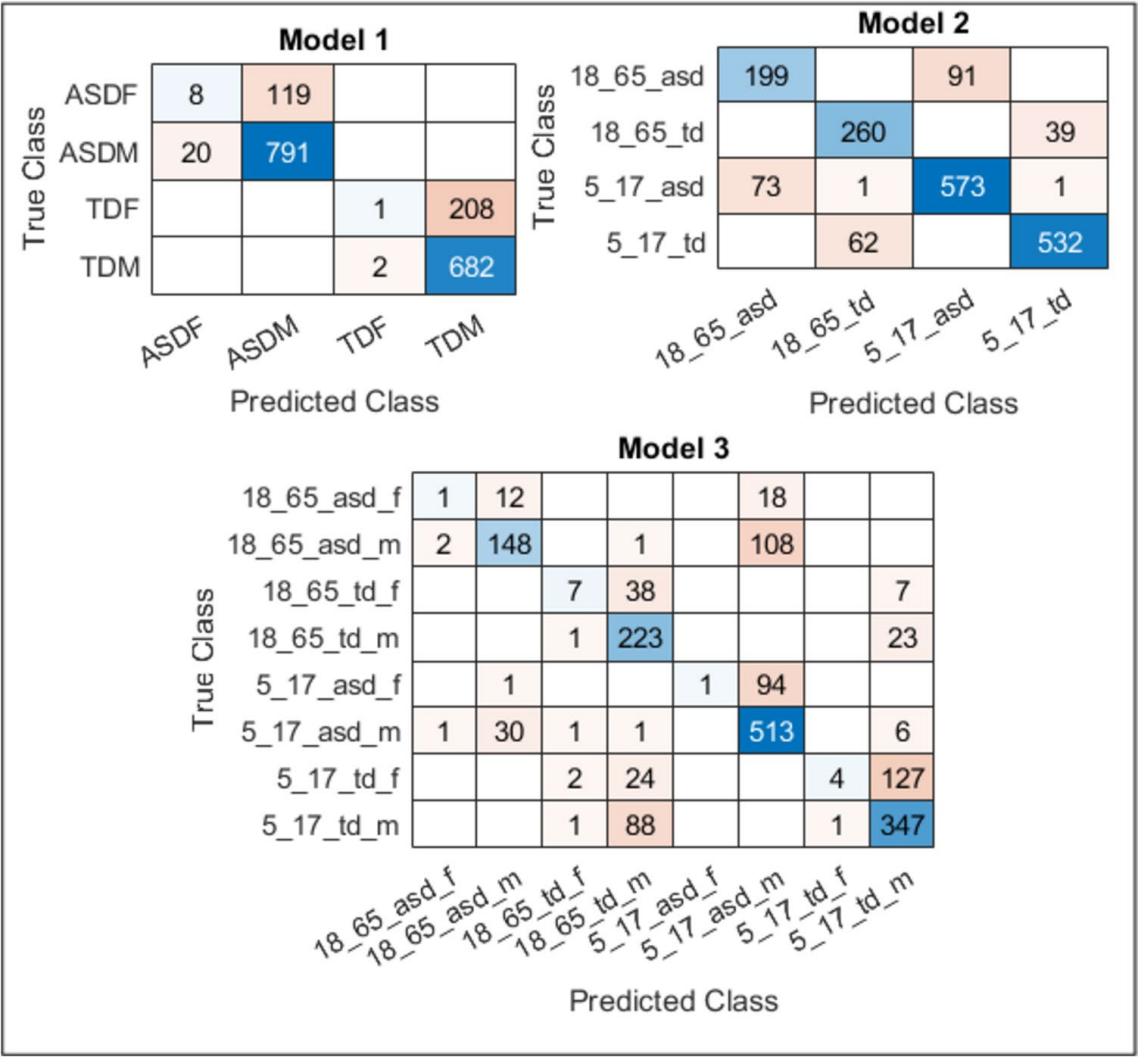
Confusion matrices for all three models

In this study, a system was developed that can contribute to ASD automatic diagnosis. CNN models, a part of this system, are designed to have the most optimal parameters through the GSO algorithm. Within this system, three CNN models were designed, and multiple classifications were performed to view the role of gender-age factors in diagnosing ASD. Gender with Model 1, age with Model 2, and both with Model 3 were highlighted. In addition, the developed system was compared to four pre-trained networks using TL. The accuracy and loss curves acquired for all three models utilized in the designed system in Fig. 7, the confusion matrices in Fig. 8, and the comparison of the results with the pre-trained networks in Table 5 are presented. According to the results given in Table 5, the accuracy rate obtained in the quadruple classification made with Model 1, which highlights the gender factor, is 80.94% and is higher than all pre-trained models designed for the same purpose. It is seen that an accuracy rate of 85.42% was achieved in the quadruple classification made with Model 2, which highlights the age factor. It is seen that the accuracy rates obtained with pre-trained models designed for the same purpose are higher than all of them. This result leads us to think that the age factor has a greater impact on the diagnosis of ASD than the gender factor. An accuracy rate of 67.94% was achieved with the octahedral classification model made with Model 3, which takes both age and gender factors into consideration. Although it seems to be less successful than Model 1 and Model 2, this result is quite high for the eight-fold classification. When compared to other eight-class classification pre-trained models implemented for the same purpose, it is seen that the highest result is obtained with Model 3. Similar comments can be made by examining missing values. With this system designed for ASD classification and diagnosis, it is seen that the effect of gender and age factors in multiple classification emerges. The results showed that all three networks outperformed the pre-trained models. In the diagnosis of ASD, the influence of the age factor seems to be more than the gender factor. With this system designed for ASD classification and diagnosis, it is seen that the influence of gender and age factor in multi-classification is revealed.

**Table 5:** Test results for all three classifications and comparison with pre-trained models

	**Model 1 (Gender)**		**Model 2 (Age)**		**Model 3 (Both)**	
**CNN Models**	**Accuracy** **(%)**	**Loss**	**Accuracy** **(%)**	**Loss**	**Accuracy** **(%)**	**Loss**
Alexnet	78.13	0.9126	81.09	0.6631	62.73	1.8453
Googlenet	77.68	1.2609	78.59	1.0999	59.32	1.9831
Resnet18	73.12	1.2083	82.46	0.8289	66.36	1.3508
Squeezenet	72.67	1.3960	79.27	1.0946	64.55	1.7776
**Proposed**	**80.94**	0.4893	**85.42**	0.3785	**67.94**	0.8418

## Conclusions and discussion

All studies on automatic diagnosis of ASD with artificial intelligence are binary classification studies. An octal classification study that takes both age and gender factors into consideration cannot be found in the literature. In this study, a deep learning system that is different from what has been done so far and is a first, as far as we know, makes quadruple and eight-fold classifications by taking age and gender factors into account and uses sMRI brain images. In this study, an estimation and classification system was designed, which, as far as we know, is different from what has been done so far, and which is a first, takes into account age and gender factors and utilizes sMRI brain images. The success and reliability of the designed system were provided by comparing it with the Alexnet, Googlenet, Resnet-18, and Squeezenet popular pre-trained networks. The model developed in this research performs better than these pre-trained models. In addition, the designed system has the feature of generalizability since the data set was acquired from the ABIDE database created by acquiring from 29 different locations, and the data set was enlarged five times by DA techniques. As a result, the accuracy rates acquired as a result of the test performed with all three CNN models designed to be utilized within the system show that the designed system has robust dynamics enough to give the highest accuracy rates.

In the future, more successful applications are planned by adding advanced (ML) algorithms like enhanced probabilistic neural network (EPNN) [74] and neural dynamic classification (NDC) algorithm [75] to a system that examines age and gender factors.

## Appendix 1

**Table:** 

**SITE**	**DEFINITON**
STANDFORD	Stanford University
KKI	Kennedy Krieger Institute
KUL	Katholieke Universiteit Leuven
LEUVEN	University of Leuven
UCD	University of California Davis
OHSU	Oregon Health and Science University
MAXMUN	Ludwig Maximilians University Munich
UCLA	University of California, Los Angeles
BNI	Barrow Neurological Institute
CALTECH	California Institute of Technology
EMC	Erasmus University Medical Center Rotterdam
GU	Georgetown University
IP	Institut Pasteur and Robert Debré Hospital
NYU	New York University Langone Medical Center
PITT	University of Pittsburgh
SDSU	San Diego State University
TRINITY	Trinity Centre for Health Sciences
UM	University of Michigan
UPSM	University of Pittsburgh School of Medicine
YALE	Yale Child Study Center
SU	Stanford University (ABIDE II)
OLIN	Olin Neuropsychiatry Research Center (ABIDE I)
ETH	Eidgenössische Technische Hochschule Zürich
TCD	Trinity College Dublin’s School of Medicine
IU	Indiana University
ONRC	Olin Neuropsychiatry Research Center (ABIDE II)
USM	University of Utah School of Medicine
CMU	Carnegie Mellon University
SBL	Social Brain Lab. BCN NIC UMC Groningen and Netherlands Institute for Neurosciences

## Data Availability

The ABIDE database, a publicly available database was used in this research.
